# Interaction Between PNPLA3 and SIRT5 Genetic Variants in Association with Liver Fibrosis Severity in Patients with Metabolic Dysfunction-Associated Steatotic Liver Disease

**DOI:** 10.3390/genes15111370

**Published:** 2024-10-24

**Authors:** Kamonchanok Moonlisarn, Pornjira Somnark, Bootsakorn Boonkaew, Chalermarat Bunchorntavakul, Pisit Tangkijvanich

**Affiliations:** 1 Center of Excellence in Hepatitis and Liver Cancer, Department of Biochemistry, Faculty of Medicine, Chulalongkorn University, Bangkok 10330, Thailand; 6570002830@student.chula.ac.th (K.M.); pornjirasomnark@gmail.com (P.S.); bootsakorn.b@gmail.com (B.B.); 2 Division of Gastroenterology, Department of Medicine, Rajavithi Hospital, Bangkok 10400, Thailand; dr.chalermrat@gmail.com

**Keywords:** metabolic dysfunction-associated steatotic liver disease (MASLD), polymorphisms, fibrosis, steatosis, *PNPLA3* rs738409, *SIRT5* rs12216101, *HSD17B13* rs6834314

## Abstract

Background/Objectives: This study evaluated the association between polymorphisms in the *PNPLA3*, *TM6SF2*, *HSD17B13*, and *SIRT5* genes and the severity of fibrosis and steatosis in metabolic dysfunction-associated steatotic liver disease (MASLD). Methods: Fibrosis and steatosis were assessed by MRE and MRI-PDFF, respectively. The polymorphisms were determined by allelic discrimination in blood samples. Results: 204 patients aged 57.0 ± 13.5 years were included. Sixty-two (30.4%) patients had significant fibrosis (≥F2). Among F2–F4 fibrosis, the *PNPLA3* rs738409 GG genotype was significantly higher than the CC + CG genotypes (44.9% vs. 21.4%, *p* = 0.001). The *SIRT5* rs12216101 GG vs. TT + TG genotypes also exhibited a similar trend (64.3% vs. 27.9%, *p* = 0.012). In multivariate analysis, the *PNPLA3* GG genotype (OR = 3.48, 95%CI: 1.50–8.06; *p* = 0.004) and *SIRT5* rs12216101 GG genotype (OR = 5.43, 95%CI: 1.32–22.33; *p* = 0.019) were independently associated with F2–F4 fibrosis. Additionally, the proportion of patients with F2–F4 fibrosis significantly increased with the number of combined risk genotypes. Among S2–S3 steatosis, the prevalence of *HSD17B13* AG + GG genotypes was higher than that of the AA genotype (37.5% vs. 23.9%, *p* = 0.048) and independently associated with moderate/severe steatosis in multivariate analysis (OR = 2.26, 95%CI: 1.14–4.49; *p* = 0.020). Conclusions: Our data indicate that the *PNPLA3* and *SIRT5* polymorphisms were independently and additively linked to significant fibrosis, while the *HSD17B13* polymorphism was associated with increased steatosis in Thai populations. These data might emphasize the importance of genetic variants in progressive MASLD.

## 1. Introduction

Metabolic dysfunction-associated steatotic liver disease (MASLD), previously termed non-alcoholic fatty liver disease (NAFLD), is one of the most prevalent chronic liver diseases (CLD) on the global scale [1]. The prevalence of MASLD is currently 25–30% of the world’s population and is notably higher in overweight or obese individuals [2,3]. The natural history of MASLD diverges from simple steatosis, steatohepatitis with or without fibrosis, cirrhosis, and the development of hepatocellular carcinoma (HCC) [4]. Additionally, MASLD has become an independent factor of cardiovascular disease that enhances the risk already present, such as type 2 diabetes (T2DM), hypertension (HT), and dyslipidemia (DLP) [5]. Thus, MASLD is now considered a clinical and economic burden, not only on liver-related complications but also associated with extrahepatic manifestations, especially among patients with advanced fibrosis/cirrhosis. Despite its increasing significance, the understanding of disease progression in patients with MASLD is not entirely known, and pharmacological treatments for MASLD are still lacking.

The pathogenesis of MASLD development involves several factors, including metabolic dysregulation, lifestyle, environmental factors, and various genetic variations [6]. Among them, single-nucleotide polymorphisms (SNPs) in the *patatin-like phospholipase domain containing 3 (PNPLA3)* gene have been documented as the primary genetic variants associated with MASLD from simple steatosis to cirrhosis and HCC [7]. Variants in the *transmembrane 6 superfamily member 2 (TM6SF2)* gene are also reported to be associated with disease progression in MASLD, regardless of *PNPLA3* polymorphism [8]. Conversely, genetic variants in *Hydroxysteroid 17-β Dehydrogenase 13 (HSD17B13)* are protective against disease progression to advanced fibrosis but might not prevent the development of steatosis [9]. More recently, SNPs at the *SIRT5* gene locus have been identified as a new risk factor associated with advanced liver disease in European patients with MASLD [10]. This variant has been shown to have a gain-of-function, increasing mitochondrial oxidative phosphorylation and oxidative stress involved in MASLD progression. However, current data regarding the role of this SNP in modulating disease severity in different MASLD populations are limited.

Accurate evaluation of liver fibrosis severity, particularly differentiating F2–F4 from mild fibrosis stages, is essential as the extent of fibrosis is a major determining factor for clinical outcomes and overall survival [11]. At present, liver biopsy is considered the gold standard for evaluating liver histopathology on MASLD. However, this invasive method has limitations as the procedure might increase the risk of complications and sampling errors [12]. Magnetic resonance elastography (MRE) and magnetic resonance imaging–proton density fat fraction (MRI-PDFF) are becoming the most reliable non-invasive techniques for assessing liver fibrosis and steatosis in MASLD, respectively [13,14]. Thus, the main aim of our cross-sectional study was to investigate whether host genetic variants were associated with the disease spectrum of fibrosis and steatosis in patients with MASLD by using an MRI-based approach as a reference method. We also explored the potential interplay between these variants and clinical parameters regarding progressive fibrosis and steatosis.

## 2. Materials and Methods

### 2.1. Patients

In this cross-sectional cohort, 204 Thai patients diagnosed with MASLD were enrolled between 2022 and 2024 at the King Chulalongkorn Memorial Hospital, Thailand. The Institute Ethics Committee approved the study (IRB No. 981/64), which was conducted according to the Declaration of Helsinki and the principles of Good Clinical Practice. Participants gave written informed consent, and blood samples were obtained from each participant after the enrollment. All participants’ anthropometric variables, including weight, height, and BMI, were measured, and clinical data were recorded.

The inclusion criteria in this study were patients aged ≥ 18 years who were diagnosed with liver steatosis based on MRI-PDFF grade ≥ 1 (specified by MRI-PDFF ≥ 5.4%) [14] and the clinical phenotype of metabolic dysfunction [1]. Exclusion criteria were as follows: (1) the presence of viral hepatitis B or C or other chronic liver diseases such as autoimmune hepatitis and Wilson’s disease; (2) the presence of complications from cirrhosis such as ascites, hepatic encephalopathy, or evidence of HCC by imaging studies; (3) presence of other disorders resulting in secondary steatosis such as human immunodeficiency virus (HIV) infection; (4) known active malignancies or severe health illness; or (5) current or historical significant alcohol intake (e.g., ≥30 g for men and ≥20 g for women).

### 2.2. Liver Stiffness and Steatosis by MRI-Based Technique

All participants undertook a non-contrast MRI-based technique for assessing liver stiffness and liver fat quantification by MRE and MRI-PDFF, respectively, using the Philips Ingenia MR imaging system at 3.0 T (Philips Healthcare, Best, The Netherlands) as previously described [15,16]. Based on meta-analysis conducted in patients with MASLD, the cut-off values for the diagnosis of liver fibrosis ≥F1, ≥F2, ≥F3, and F4 by MRE were 2.6, 3.0, 3.6, and 4.7 kPa, respectively [13]. Regarding the assessment of MRI-PDFF, the cut-off points for diagnosing liver steatosis grades ≥S1, ≥S2, and ≥S3 were 5.4%, 15.4%, and 20.4%, respectively [14].

### 2.3. DNA Preparation and SNP Analysis

Genomic DNA was extracted from 100 µL of peripheral blood mononuclear cells (PBMCs) using phenol-chloroform-isoamyl alcohol isolation, DNA quality was assessed by spectrophotometer (NanoDrop 2000c, Thermo Scientific, Waltham, MA, USA), and the samples were stored at −70 °C until analysis.

The targeted SNPs, including *PNPLA3* rs738409, *TM6SF2* rs58542926, *HSD17B13* rs6834314, and *SIRT5* rs12216101, were assessed using the real-time PCR protocol based on TaqMan assays. The reaction mixture included 4 µL of 2.5 × master mix (5 PRIME, Hamburg, Germany), 0.25 µL of 40X primer and probe mixture *TaqMan* SNP Genotyping Assay (assay ID: C_7241_10, Applied Biosystems, Waltham, MA, USA), 50–100 ng of genomic DNA, and nuclease-free water, bringing the final volume to 10 µL. Following previously described techniques [17], real-time PCR was performed on a QuantStudio3 Real-time PCR system (Applied Biosystems, USA). Briefly, the procedure included an initial denaturation at 95 °C for 10 min, followed by 50 amplification cycles consisting of denaturation at 92 °C for 10 s and annealing/extension at 60 °C for 1 min. Fluorescent signals (FAM and VIC) were utilized at the end of each cycle. Positive and negative controls were included in every experiment to ensure accurate data interpretation. The allelic discrimination plot was analyzed by the QuantStudio™ 3 Real-Time PCR System (Thermo Fisher Scientific, USA).

### 2.4. Statistical Analyses

Statistical analyses were conducted using the IBM SPSS software version 23.0 (IBM, Chicago, IL, USA). Continuous variables are presented as mean ± standard deviation (SD), and categorical variables are presented as frequencies and percentages. At the same time, the comparisons between groups were performed by analysis of variance and Student’s *t*-test or the nonparametric Mann–Whitney U test when appropriate. The odds ratio (OR) with a 95% confidence interval (CI) between groups was also determined. Spearman’s rank test was applied to evaluate the correlation of various parameters. Univariate and multivariable analyses were undertaken using binary logistic regression to determine parameters related to fibrosis F2–F4 or steatosis S2–S3. A *p*-value < 0.05 was interpreted as statistically significant.

## 3. Results

### 3.1. Patient Characteristics

The clinical and laboratory features of 204 patients according to fibrosis stages [no/mild fibrosis (F0–F1, *n* = 142) and moderate/severe fibrosis (F2–F4, *n* = 62)] are shown in Table 1. The F2–F4 group had a greater mean age and higher distribution of T2DM and hypertension (HT) than patients with F0–F1 fibrosis (all *p* < 0.001). Compared to the F0–F1 group, patients in the F2–F4 group had higher serum aspartate aminotransaminase (AST, *p* < 0.001), alanine aminotransaminase (ALT, *p* = 0.026), and mean MRE measurements but had a lower platelet count and MRI-PDFF value (both *p* < 0.001). However, there was no difference in gender distribution, BMI, presence of dyslipidemia (DLP), or other clinical parameters between the groups (all *p* > 0.05).

### 3.2. Distributions of the SNPs and Association with Fibrosis and Steatosis

In this cohort, the frequencies of *PNPLA3* rs738409 (CC/CG/GG) of the whole cohort were 52 (25.5%)/74 (36.3%)/78 (38.2%), while the distributions of *TM6SF2* rs58542926 (CC/CT/TT) were 155 (76.5%)/39 (19.1%)/9 (4.4%). The frequencies of *HSD17B13* rs6834314 (AA/AG/GG) were 92 (45.1%)/89 (43.6%)/23 (11.3%), and the distributions of *SIRT5* rs12216101 (TT/TG/GG) were 112 (54.9%)/78 (38.2%)/14 (6.9%). The distributions of each SNP according to the fibrosis groups are shown in Table 1.

**Figure 1:**
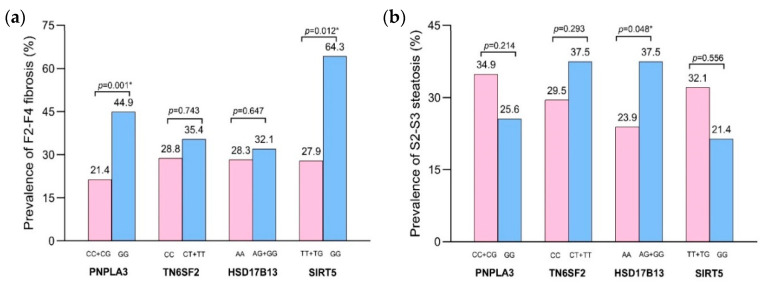
Prevalence of the SNPs in this study: (**a**) patients with F2–F4 fibrosis; (**b**) patients with S2–S3 steatosis. * *p* < 0.05.

Patients with F2–F4 fibrosis had a significantly higher frequency of the *PNPLA3* GG genotype than the CC + CG genotypes (44.9% vs. 21.4%, *p* = 0.001). A similar trend was observed for the *SIRT5* GG vs TT + TG genotypes (64.3% vs. 27.9%, *p* = 0.012). However, the frequencies of *TM6SF2* CT + TT vs. CC genotypes were not significant (35.4% vs. 28.8%, *p* = 0.743), which was similar to the distributions of *HSD17B13* AG + GG vs. AA genotypes (32.1% vs. 28.3%, *p* = 0.647) (Figure 1a). Notably, female individuals with the *PNPLA3* GG genotype tended to have a higher mean MRE value than male patients harboring the same genotype (3.6 ± 1.7 vs. 2.9 ± 1.1, *p* = 0.074). However, there was no significant difference in MRE levels between female and male individuals who carried CC + CG genotypes (2.7 ± 1.2 vs. 2.7 ± 1.1, *p* = 0.908). Regarding the *SIRT5* variants, there was no significant difference between female and male patients who carried the GG genotype (3.5 ± 1.3 vs. 3.1 ± 0.8, *p* = 0.473) or the TT + TG genotypes (3.0 ± 1.4 vs. 2.8 ± 1.1, *p* = 0.301).

The genotype frequencies of SNPs associated with steatosis grades were also investigated. Patients with grade 2–3 steatosis (S2–S3) had an increased frequency of the *HSD17B13* AG + GG genotype than the AA genotypes (37.5% vs. 23.9%, *p* = 0.048). However, the other polymorphisms were not significantly associated with the severity of steatosis: *PNPLA3* GG vs. CC + CG genotypes (25.6% vs. 34.9%, *p* = 0.214), *TM6SF2* CT + TT vs. CC genotypes (37.5% vs. 29.5%, *p* = 0.293), and *SIRT5* GG vs. TT + TG genotypes (21.4% vs. 32.1%, *p* = 0.556) (Figure 1b).

### 3.3. Additive Effects of the SNPs Associated with Fibrosis

**Figure 2:**
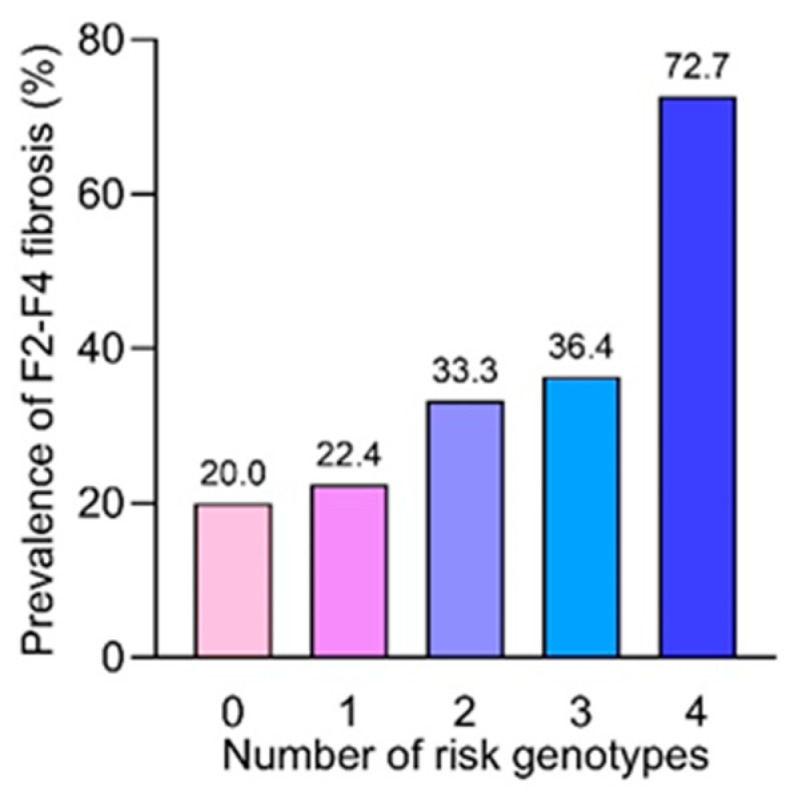
Additive effects of *PNPLA3* and *SIRT5* genetic variants on the risk of F2–F4 fibrosis. Risk genotypes were counted and summed in an additive model at *PNPLA3* (by coding 0, 1, and 2 for CC, CG, and GG genotypes, respectively) and at *SIRT5* (by coding 0, 1, and 2 for TT, TG, and GG genotypes, respectively).

The combined effect of the risk genotypes, including the PNPLA3 GG genotype and the SIRT5 GG genotype, in association with progressive fibrosis, was further investigated. In this regard, we considered risk genotypes at PNPLA3 rs738409 (by coding 0, 1, and 2 for CC, CG, and GG genotypes, respectively) and at SIRT5 rs12216101 (by coding 0, 1, and 2 for TT, TG, and GG genotypes, respectively) for each patient and examined whether these variants employed an additive effect on the severity of fibrosis. Figure 2 demonstrates the percentage of patients with F2–F4 fibrosis harboring different numbers of risk genotypes. In this study, 20.0%, 22.4%, 33.3%, 36.4%, and 72.7% of patients with F2–F4 fibrosis carried 0, 1, 2, 3, and 4 risk genotypes, respectively (*p* = 0.008, chi-square test for trend analysis). Thus, the percentage of patients with F2–F4 fibrosis increased significantly along with the accumulated numbers of the risk genotypes. Multiple logistic regression showed that there were additive effects of the accumulated risk genotypes on the presence of F2–F4 fibrosis as follows: 0 (reference group, OR = 1), 1 (OR = 4.67; 95%CI: 1.04–21.01; *p* = 0.045), 2 (OR = 5.33; 95%CI: 1.28–22.21; *p* = 0.021), 3 (OR = 9.24; 95%CI: 2.18–39.25; *p* = 0.003), and 4 [OR = 10.67; 95%CI: 2.15–52.85; *p* = 0.004].

### 3.4. Factors Associated with Significant Fibrosis and Steatosis

We investigated whether the parameters were independently associated with significant fibrosis (≥F2) and steatosis (≥S2). For predicting fibrosis, selected parameters in the univariate analysis included age, T2DM, HT, AST, platelet count, steatosis grade, and *PNPLA3* and *SIRT5* variants. In multivariate analysis, T2DM, platelet count, AST, *PNPLA3,* and *SIRT5* were independently associated with significant fibrosis (Table 2). Regarding factors associated with steatosis, the univariate analysis revealed age, BMI, ALT, fibrosis stage, and *HSD17B13* variant, whereas only age, ALT, and *HSD17B13* variant were selected in the multivariate analysis (Table 3).

## 4. Discussion

MASLD is now considered the most common chronic liver disease worldwide and is regarded as a multisystem disorder with a broad spectrum of clinical phenotypes. Thus, understanding its natural history regarding the extent of fibrosis and steatosis is necessary to stratify patients’ risks for clinical management. Besides metabolic risk factors, several genetic variants have been identified to influence the risk of MASLD progression. In this cross-sectional study of well-characterized patients with MASLD, our multivariate analysis indicated the significant associations between fibrosis severity and *PNPLA3* rs738409 and *SIRT5* rs12216101. Additionally, we demonstrated an interaction of genetic variants in the development of fibrosis in MASLD. Specifically, we showed that carrying the *PNPLA3* GG with *SIRT5* GG was associated with an increased risk of significant fibrosis by MRE. Regarding steatosis, our data showed that *HSD17B13* rs6834314 GA + GG was an independent factor associated with moderate/severe steatosis assessed by MRI-PDFF. These data highlight the importance of genetic variants in progressive disease; thus, identifying risk variants could help detect significant fibrosis or steatosis among subjects with MASLD.

Current advances have established that genetic alteration variants are crucial factors responsible for the development and progression of MASLD [7,18]. Genetic variants involving SNPs in different genes have been uncovered through genome-wide association studies (GWASs) and exome-wide association studies. *PNPLA3*, a trans-membrane protein belonging to the patatin-like phospholipase family, is mainly expressed in the liver and exhibits hydrolase activity against triglycerides [19]. It has been shown that the loss-of-function *PNPLA3* rs738409 variant is associated with reduced lipolysis, thereby leading to increased intrahepatic triglyceride accumulation and inflammation [20]. In this study, our data underlined the role of the *PNPLA3* GG genotype in association with significant fibrosis, which aligned with previous meta-analysis data indicating an increased risk of this variant on progressive fibrosis in MASLD [21]. The molecular mechanisms by which the *PNPLA3* variant favors fibrogenesis are not entirely clarified. However, it has been shown in in vitro models that primary human hepatic stellate cells (HSCs) harboring this polymorphism exhibit enhanced pro-fibrogenic features, resulting in increased inflammation and fibrogenesis, leading to disease progression [22]. In our study, this association of the variant remained significant in multivariate analysis after considering other potential cofactors, such as T2DM, indicating an essential role of this variant in determining severe fibrosis. Of note, female individuals with the *PNPLA3* GG genotype tended to have higher MRE values than male patients harboring the same genotype. These findings were in line with previous data supporting the role of the female gender in interacting with the variant via estrogen receptor-α in the susceptibility and severity of MASLD [23].

Although significant associations between the *PNPLA3* GG carriage and MASLD have been documented in several studies, studies regarding the association between the *SIRT5* rs12216101 variant and advanced liver disease in patients with MASLD are scarce. Sirtuins (SIRTs), a family of nicotinamide adenine dinucleotide (NAD+)-dependent deacetylases, are involved in several critical signaling pathways and biological processes, such as cellular metabolism, inflammation, and lifespan regulation [24]. Typically, the mitochondrial SIRT5 modulates the activity of mitochondrial enzymes in response to fasting and calorie restriction. However, the gain-of-function *SIRT5* rs12216101 polymorphism was linked to enhanced sirtuin activity, which led to susceptibility to cardiometabolic disorders, such as developing carotid plaque, a surrogate marker of atherosclerosis [25]. Several proposed mechanisms of the *SIRT5* variant included upregulated mitochondrial oxidative phosphorylation, increased reactive oxygen species, and interaction with various metabolic risk factors [10,25]. In an animal model, the over-expressed intrahepatic SIRT5 protein was linked to mitochondrial dysfunction and upregulated pro-inflammatory mediators, leading to the activation of fibrogenesis and advanced steatotic liver disease [26]. Additionally, the *SIRT5* variant was associated with more severe histological features of liver disease, such as inflammation, steatohepatitis, and significant fibrosis in the Western population with MASLD [10]. Notably, the *SIRT5* polymorphism could influence alternative splicing and possibly induce the expression of SIRT5 isoform 4 (SIRT5iso4). In this study, our results indicated that the *SIRT5* GG genotype was linked to a significantly greater distribution in the F2–F4 group versus the F0–F1 group of MASLD. In the multivariate regression analysis, the GG genotype remained an independent predictive factor associated with advanced fibrosis, similar to the above-mentioned report.

Given the independent mechanisms involved in progressive fibrosis by *PNPLA3* rs738409 and *SIRT5* rs12216101, these two variants might synergistically increase the risk of advanced fibrosis in MASLD. In this regard, according to MRE measurement, our data confirmed the additive effects of *PNAPLA3* and *SIRT5* on the increased severity of fibrosis. These findings might imply the potential interplay between these two genetic variants, as the risk conferred by the *PNPLA3 GG* genotype appeared to be augmented by the *SIRT5* GG carriage. Although the mechanism by which combined variants lead to progressive fibrosis is not yet completely understood, it is speculated that they might synergistically induce oxidative stress and the production of several proinflammatory mediators in predisposed increased intrahepatic fat, which modulates the development of hepatic inflammation and fibrogenesis following the multiple-hit pathogenesis of MASLD [27]. Thus, combining assays of the *PNPLA3* and *SIRT5* GG genotypes as genetic risk factors may have clinical applications, as they could permit the discrimination of patients with a high likelihood of developing severe liver disease.

The pathophysiological role of the *HSD17B13* polymorphisms on MASLD is not yet fully understood and requires further elucidation. Our study also found that the *HSD17B13* AG/GG genotypes were not associated with advanced fibrosis but were significantly associated with the degree of steatosis. In general, *HSD17B13* encodes for hepatic retinol-dehydrogenase essential for regulating hepatic lipid homeostasis. The rs6834314 variant is associated with losing its enzymatic activity despite regular protein expression and localization, which might be involved in the pathogenesis of steatosis [28]. However, the role of the *HSD17B13* variant in association with increased steatosis is inconsistent among reports [18]. For example, a recent study in Japan demonstrated that the *HSD17B13* rs6834314 AG/GG variants were associated with increased steatosis grading but exhibited an attenuated effect of *PNPLA3* on advanced hepatic fibrosis [29]. In a cohort of multi-ethnic Asian patients with MASLD, however, the *HSD17B13* rs6834314 variant was inversely associated with steatosis and steatohepatitis, as well as linked to lower incidence of adverse hepatic outcomes in long-term follow-up [30].

Notably, our data did not verify the role of the *TM6SF2* polymorphism in correlation with fibrosis or steatosis severity. The *TM6SF2* gene, predominately expressed in the liver, encodes proteins responsible for lipid metabolism by modulating hepatic triglyceride secretion [31]. The *TM6SF2* rs5842926 variant is associated with enriched intrahepatic triglyceride and lower very-low-density lipoprotein (VLDL) secretion. Interestingly, the genetic variant confers a greater risk of developing steatosis and fibrosis but could protect against cardiovascular disease [32]. Although the *TM6SF2* polymorphism might increase the risk of MASLD, its connection toward predisposition to entire disease spectra is unclear and presents conflicting information. For example, the *TM6SF2* SNP could affect fibrosis progression in Western patients but did not affect advanced fibrosis or cirrhosis in Asian subjects [33,34]. Thus, these data indicate that the differences in ethnicities or clinical features of the study cohorts might influence the association among studies. Moreover, a recent meta-analysis also showed that this SNP tends to have a stronger association with progressive fibrosis or steatosis in children than in adults with MASLD [35].

This study might have some limitations, including being a cross-sectional cohort, which recruited a relatively small number of patients with F2–F4 fibrosis, which could lead to inadequate statistical power as a type II error. This limitation might reflect the smaller distribution of significant fibrosis to cirrhosis in real-life settings. For example, a European study conducted in a real-world cohort showed that the frequency of F2–F4 fibrosis was approximately 30–35% [36], comparable to our report. Moreover, this study was conducted in a single tertiary referral center, potentially subject to selection bias, and whether our results could be utilized in the general population is unclear. Additionally, our cohort recruited only Thai patients, which might not apply to other ethnic populations. Finally, liver biopsy was not performed to assess fibrosis and steatosis in our cohort because of its invasive method, as mentioned previously. Instead, we applied MRI-based modalities, including MRE and MRI-PDFF, which were more feasible in clinical settings. Despite the limitations, our results suggested that the *SIRT5* genetic variant is an independent predictive marker of advanced fibrosis in Thai individuals. This has confirmed and extended the observations of previously published data in Western patients that this variant may represent a novel genetic biomarker in MASLD. Currently, several genetic polymorphisms related to MASLD have gained increasing attention for their possible use as disease-associated variants in precision medicine. In this regard, there is a need for further investigations to clarify the precise molecular mechanisms of these SNPs, particularly *SIRT5* rs12216101, which might pave the way for the development of new therapy in patients with MASLD.

## 5. Conclusions

In conclusion, our study indicated that the *PNPLA3* and *SIRT5* polymorphisms, individually and in combination, might influence the severity of fibrosis in patients with MASLD. Moreover, the *HSD17B13* variant was significantly associated with the degree of steatosis. These data could support the clinical utility of genetic genotyping in identifying patients with MASLD who are at increased risk of unfavorable outcomes. Further studies with long-term follow-up are needed to confirm these observations and elucidate how these variants influence the development of severe MASLD in individuals with different ethnic and clinical backgrounds.

## Figures and Tables

**Table 1:** Clinical characteristics of the patients.

Characteristics	MASLD (F0–F1) (*n* = 142)	MASLD (F2–F4) (*n* = 62)	*p*-Value
Age (years)	54.3 ± 13.5	63.0 ± 11.4	<0.001 *
Gender			0.450
Male	75 (52.8)	29 (46.8)	
Female	67 (47.2)	33 (53.2)	
Body mass index (kg/m^2^)			0.745
<25.0	39 (27.5)	16 (25.8)	
25.0–29.9	69 (48.6)	28 (45.2)	
>30.0	34 (23.9)	18 (29.0)	
Presence of type 2 diabetes	32 (22.5)	32 (51.6)	<0.001 *
Presence of hypertension	50 (35.2)	39 (62.9)	<0.001 *
Presence of dyslipidemia	52 (36.6)	20 (32.3)	0.633
Hemoglobin (g/dL)	14.1 ± 1.5	13.5 ± 1.7	0.127
White blood count (10^3^/µL)	6.7 ± 2.1	7.0 ± 2.1	0.910
Platelet count (10^3^/µL)	261.5 ± 63.1	213.2 ± 82.2	<0.001 *
Total bilirubin (mg/dL)	0.7 ± 0.3	0.8 ± 0.3	0.746
Serum albumin (g/dL)	4.4 ± 0.2	4.3 ± 0.5	0.139
Aspartate aminotransferase (IU/L)	24.8 ± 9.3	37.0 ± 18.5	<0.001 *
Alanine aminotransferase (IU/L)	34.9 ± 21.2	39.8 ± 25.0	0.026 *
Alkaline phosphatase (IU/L)	70.6 ± 22.7	75.7 ± 28.9	0.161
Magnetic resonance elastography (kPa)	2.2 ± 0.3	4.4 ± 1.3	<0.001 *
Proton density fat fraction (%)	13.0 ± 7.7	10.2 ± 6.6	0.014*
*PNPLA3* rs738409 (CC/CG/GG)	41 (28.8)/58 (40.8)/43 (30.3)	11 (17.7)/16 (25.8)/35 (56.5)	0.002 *
*TM6SF2* rs58542926 (CC/CT/TT)	111 (78.2)/28 (19.7)/3 (2.1)	45 (72.6)/11 (17.7)/6 (9.7)	0.053
*HSD17B13* rs6834314 (AA/AG/GG)	66 (46.5)/59 (41.5)/17 (12.0)	26 (41.9)/30 (48.4)/6 (9.7)	0.650
*SIRT5* rs12216101 (TT/TG/GG)	80 (56.3)/57 (40.1)/5 (3.5)	32 (51.6)/21 (33.3)/9 (14.5)	0.006 *

Data expressed as mean ± SD or *n* (%), * *p*-value < 0.05.

**Table 2:** Factors associated with significant fibrosis (F2–F4).

		Univariate Analysis		Multivariate Analysis	
		OR (95%CI)	*p*-Value	OR (95%CI)	*p*-Value
Age (years)	≥60 vs. <60	2.23 (1.21–4.10)	0.010 *	1.85 (0.72–4.72)	0.200
Gender	Male vs. Female	1.27 (0.70–2.32)	0.428		
BMI (kg/m^2^)	≥25 vs. <25	1.09 (0.55–2.14)	0.806		
Type 2 Diabetes	Yes vs. No	3.67 (1.94–6.92)	<0.001 *	4.05 (1.75–9.40)	0.001 *
Hypertension	Yes vs. No	3.12 (1.68–5.80)	<0.001 *	2.01 (0.85–4.78)	0.113
Dyslipidemia	Yes vs. No	0.82 (0.44–1.55)	0.549		
Hemoglobin (g/dL)	<13.0 vs. ≥13.0	0.58 (0.22–1.49)	0.254		
Platelet count (10^9^/L)	<150 vs. ≥150	6.05 (1.96–18.70)	0.002 *	6.28 (1.72–23.01)	0.006 *
Total bilirubin (mg/dL)	≥0.7 vs. <0.7	0.73 (0.28–1.92)	0.527		
Serum albumin (g/dL)	<4.3 vs. ≥4.3	2.10 (0.95–4.24)	0.079		
Aspartate aminotransferase (IU/L)	≥40 vs. <40	1.08 (1.04–1.11)	<0.001 *	13.40 (3.97–45.17)	<0.001 *
Alanine aminotransferase (IU/L)	≥40 vs. <40	1.10 (0.99–1.02)	0.163		
Alkaline phosphatase (IU/L)	≥100 vs. <100	1.44 (0.30–6.94)	0.650		
Liver steatosis grade	S2 + S3 vs. S1	0.47 (0.24–0.95)	0.037 *	0.58 (0.21–1.63)	0.299
*PNPLA3* rs738409	GG vs. CC + CG	2.98 (1.61–5.53)	0.001 *	3.48 (1.50–8.06)	0.004*
*TM6SF2* rs58542926	CT + TT vs. CC	1.35 (0.68–2.69)	0.388		
*HSD17B13* rs6834314	AA vs. AG + GG	1.20 (0.66–2.20)	0.549		
*SIRT5* rs12216101	GG vs. TT + TG	4.65 (1.49–14.52)	0.008 *	5.43 (1.32–22.33)	0.019 *

OR = odds ratio, CI = confidence interval, * *p* < 0.05.

**Table 3:** Factors associated with moderate/severe steatosis (S2–S3).

		Univariate Analysis		Multivariate Analysis	
		OR (95%CI)	*p*-Value	OR (95%CI)	*p*-Value
Age (years)	≥60 vs. <60	0.30 (0.16–0.57)	<0.001 *	0.43 (0.22–0.87)	0.018 *
Gender	Male vs. Female	0.88 (0.49–1.60)	0.679		
BMI (kg/m^2^)	≥25 vs. <25	2.56 (1.19–5.48)	0.016 *	1.80 (0.79–4.11)	0.162
Type 2 Diabetes	Yes vs. No	0.99 (0.52–1.88)	0.980		
Hypertension	Yes vs. No	0.57 (0.31–1.05)	0.073		
Dyslipidemia	Yes vs. No	0.69 (0.37–1.31)	0.258		
Hemoglobin (g/dL)	<13.0 vs. ≥13.0	1.90 (0.57–6.29)	0.293		
Platelet count (10^9^/L)	<150 vs. ≥150	7.13 (0.91–55.55)	0.061		
Total bilirubin (mg/dL)	≥0.7 vs. <0.7	1.03 (0.39–2.67)	0.951		
Serum albumin (g/dL)	<4.3 vs. ≥4.3	0.71 (0.32–1.32)	0.114		
Aspartate aminotransferase (IU/L)	≥40 vs. <40	1.16 (0.49–2.75)	0.745		
Alanine aminotransferase (IU/L)	≥40 vs. <40	3.03 (1.62–5.66)	0.001 *	2.85 (1.42–5.73)	0.003 *
Alkaline phosphatase (IU/L)	≥100 vs. <100	0.49 (0.36–8.43)	0.492		
Liver steatosis grade	S2 + S3 vs. S1	0.47 (0.24–0.95)	0.037 *	0.47 (0.22–1.02)	0.056
*PNPLA3* rs738409	GG vs. CC + CG	0.64 (0.34–1.20)	0.167		
*TM6SF2* rs58542926	CT + TT vs. CC	1.44 (0.73–2.83)	0.297		
*HSD17B13* rs6834314	AA vs. AG + GG	1.91 (1.03–3.52)	0.039 *	2.26 (1.14–4.49)	0.020 *
*SIRT5* rs12216101	GG vs. TT + TG	0.58 (0.16–2.14)	0.411		

OR = odds ratio, CI = confidence interval, * *p* < 0.05.

## Data Availability

The original contributions presented in the study are included in the article, further inquiries can be directed to the corresponding author.
